# Supplementary material for: Multiparametric biophysical profiling of red blood cells in malaria infection
Source: Commun Biol. 2021 Jun 8;4:697. doi: 10.1038/s42003-021-02181-3 (PMC8187722; doi:10.1038/s42003-021-02181-3)
Supplement: Supplementary file 2 — Supplementary Information [file 42003_2021_2181_MOESM2_ESM.pdf]

**Supplementary information: Figures**

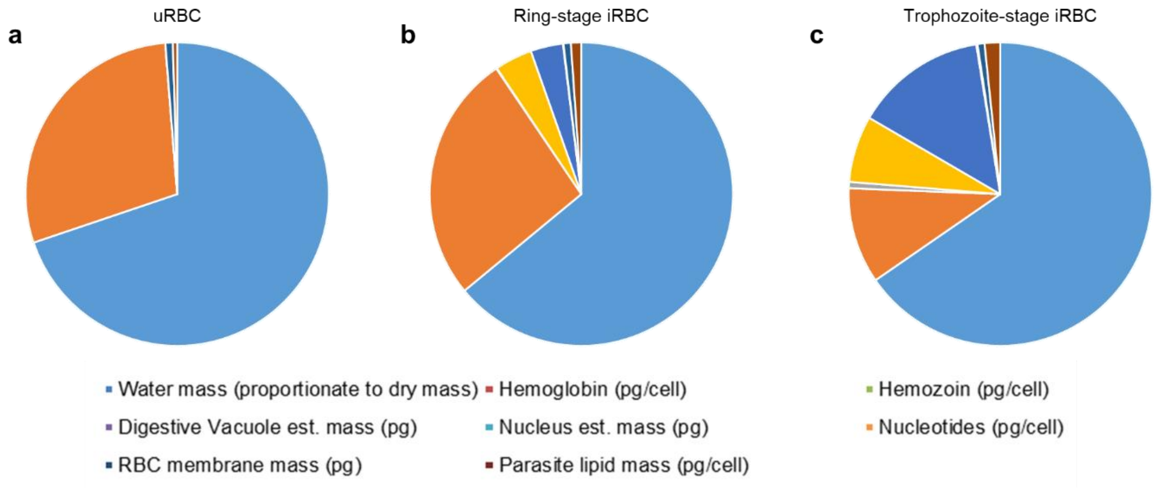


**Figure S1.** Estimated single cell biophysical parameters of erythrocytes undergoing *Plasmodium falciparum* infection, estimated by integrating various quantitative biophysical measurements and models. Key denotes various cellular components shown in pie charts.

**(a)** Uninfected erythrocyte (uRBC). **(b)** Ring-stage iRBC. **(c)** Trophozoite-stage iRBC.

Masses estimated from spectral measurements, published by Serebrennikova *et al* in J. Theoretical Biology. *Quantitative analysis of morphological alterations in Plasmodium falciparum infected red blood cells through theoretical interpretation of spectral measurements* (2010); volumes estimated by quantitative phase spectroscopy (QPS), published by Rinehart *et al* in Sci. Rep. *Hemoglobin consumption by P. falciparum in individual erythrocytes imaged via quantitative phase spectroscopy.* (2016).

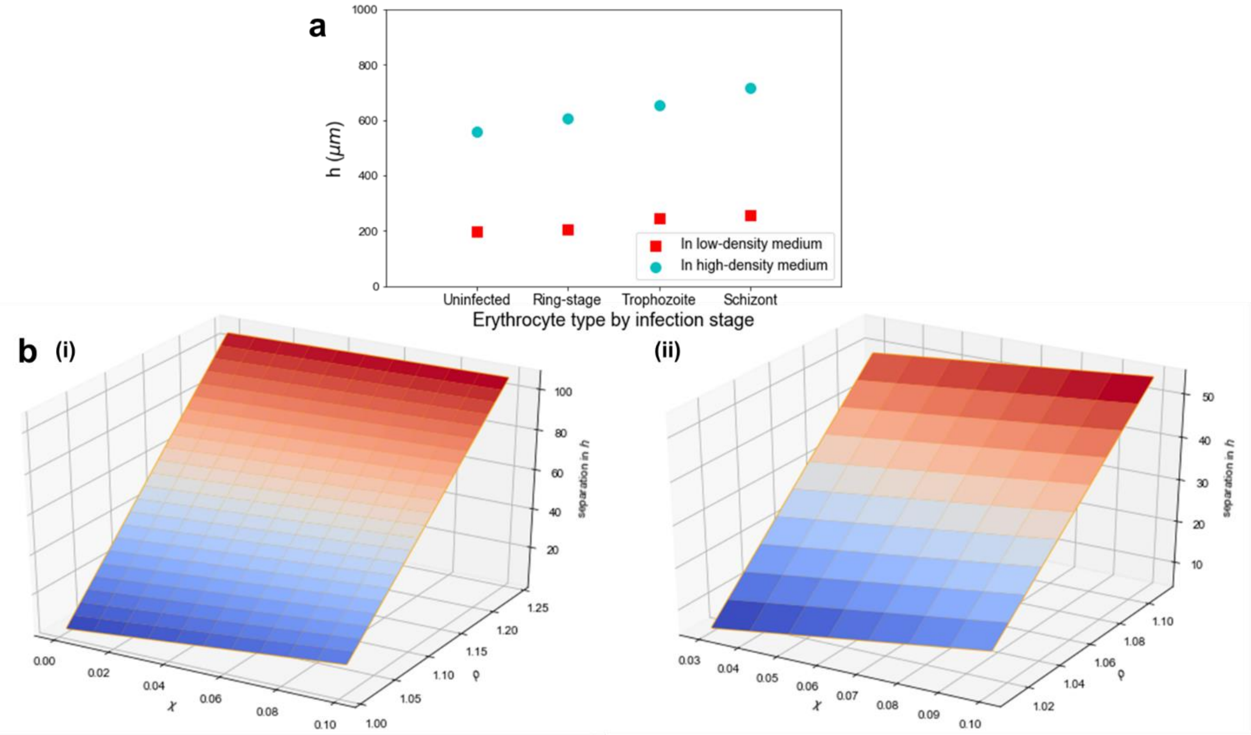


**Fig. S2. (a)** Predicted heights of erythrocytes of different infection stages in low-density medium (5 µm separation between ring-stage and uninfected) and high-density medium (53 µm separation between ring-stage and uninfected) under a fixed set of medium conditions other than density.

**(b)** Modelling predicted height separation between uninfected RBCs and ring-stage infected RBCs. **(i)** covers the full density and magnetic susceptibility ranges we calculated in our theoretical model, and **(ii)** covers the physically realistic and achievable ranges of medium density and magnetic susceptibility with the materials we used.

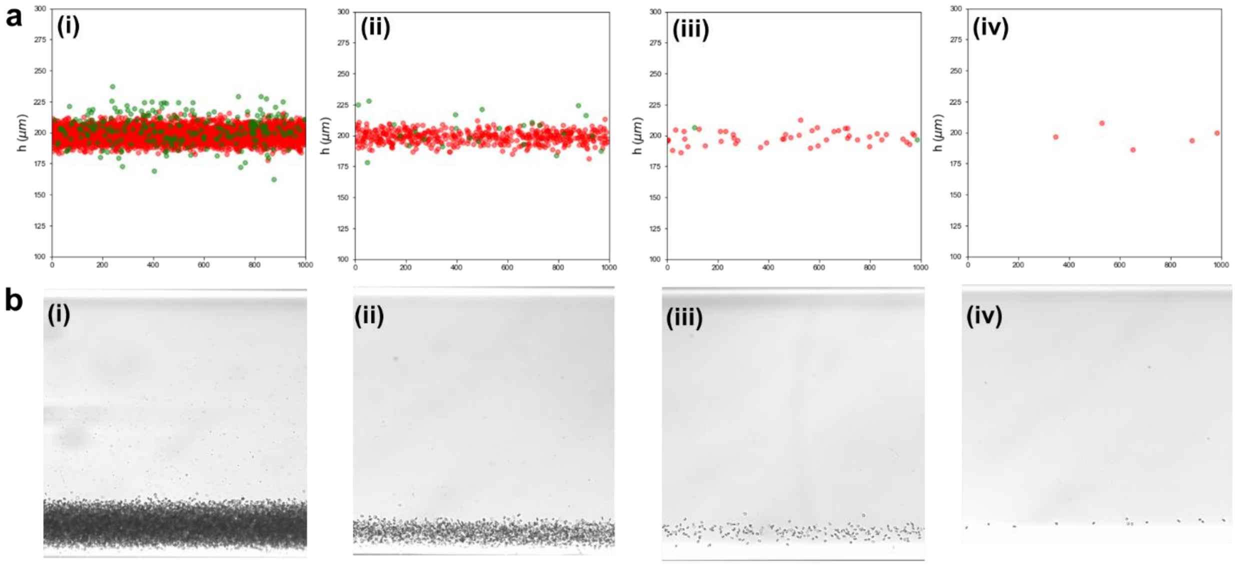


**Figure S3. (a)** Simulation of ring-stage synchronized infected culture at 5% parasitemia in a low-density medium (1.01 g/mL) at low magnetic susceptibility ($1.12{\cdot}10^4$), as in Fig. 2e,f, at various dilution factors: (i) Diluted from whole blood equivalent by (1:10). (ii) Diluted from whole blood equivalent by (1:100). (iii) Diluted from whole blood equivalent by (1:1000). (iv) Diluted from whole blood equivalent by (1:10,000).

**(b)** Images of levitated whole blood in a low-density medium (1.01 g/mL) at low magnetic susceptibility ($1.12{\cdot}10^4$), as in **Fig. 2e,f,** at the corresponding dilution factors.

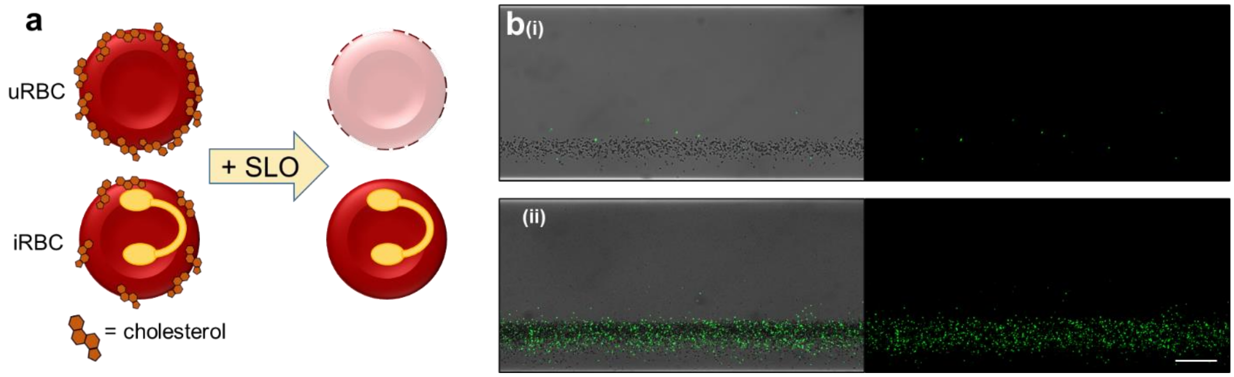


**Figure S4.** Selective lysis with streptolysin O (SLO), a streptococcal pore-forming toxin that targets cholesterol-rich cell membranes. As in **(a)**, uRBC have relatively cholesterol-rich cell membranes than iRBC whose membrane cholesterol is partially depleted due to recruitment for parasitic processes. This makes uRBC more susceptible to cholesterol-targeting lysis than iRBC. Thus, our protocol (based on Jackson *et al,* 2007), involves treatment of infected cultures with SLO, which results in selective lysis of uRBC, and concentration of the cells. The result of this, as seen in **(b)**, is a population of erythrocytes significantly enriched for iRBC, with ghosted uRBC occurring in the solution but not interfering with levitation imaging or analysis. **(b)(i)** shows an example of normal culture in levitation, with a small (<5%) percentage of iRBC, while **(ii)** shows an example of SLO-treated culture, enriched by a factor of ~15 to be majority iRBC, confirmed with acridine orange staining and fluorescence imaging. Scale bar: 200 µm.

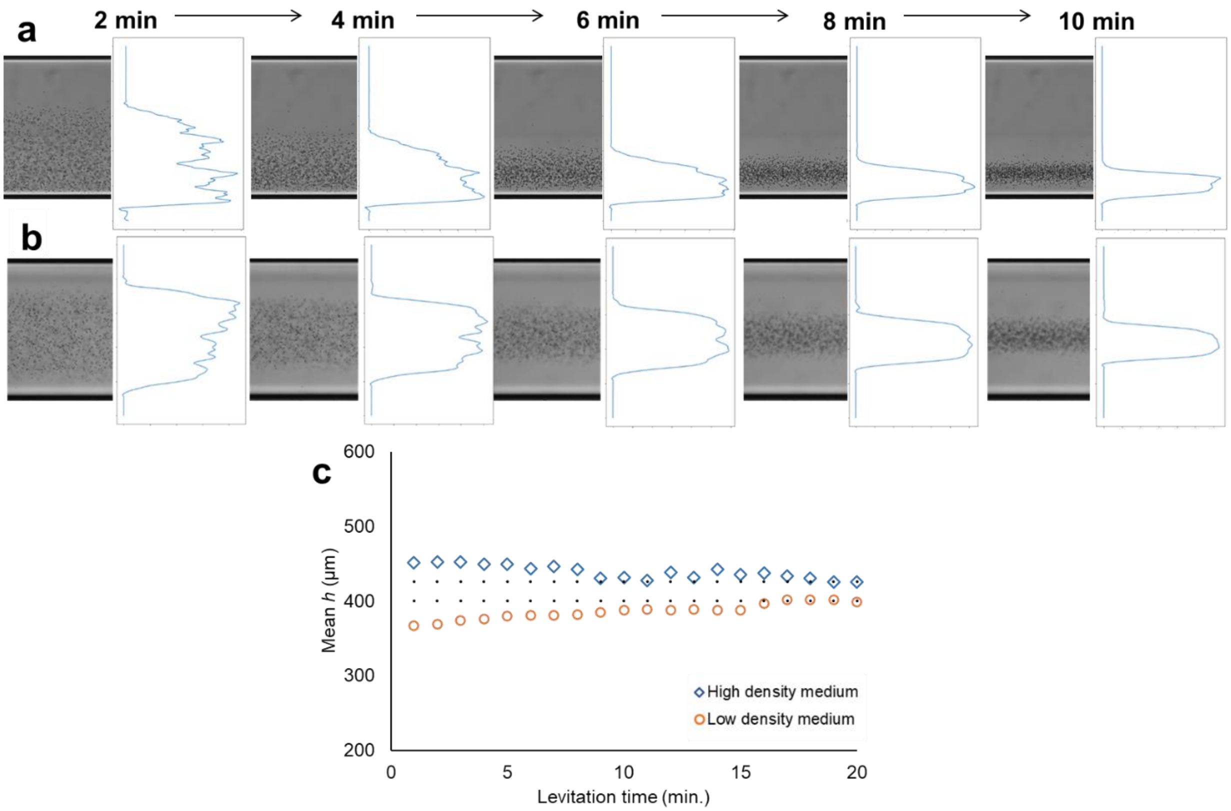


**Figure S5.** Time to levitation height equilibrium. **(a)** Images and height distributions of a typical RBC population in levitation over time, since insertion into device (in the case of low-density medium). **(b)** Images and height distributions of a typical RBC population in levitation over time, since insertion into device (in the case of high-density medium). **(c)** Mean height of cells in levitation chamber over time from sample insertion, in both cases: cells start to disperse, begin to sediment, and come to equilibrium within 15-20 minutes.

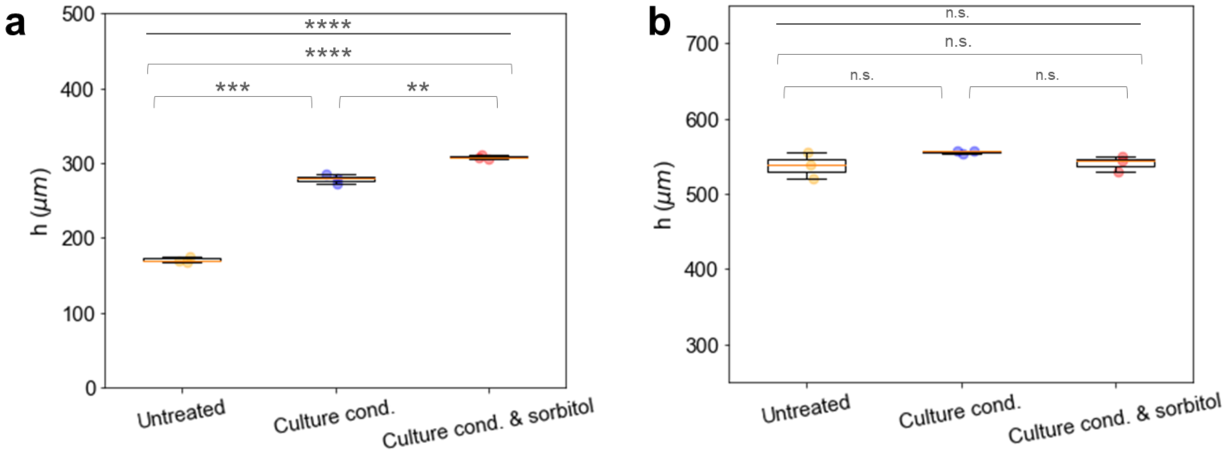


**Figure S6.** Comparison of control (uninfected) RBCs in normal storage (4 degrees Celsius) with uninfected RBCs kept in standard 3D7 culture conditions (incubated in culture medium) for 72 hours, as well as uninfected RBCs kept in standard culture conditions 72 hours then additionally subjected to the sorbitol synchronization protocol.

The above three culture conditions were sampled and levitated in (**a**) the low-density medium levitation condition, and in (**b)** high-density medium levitation condition.

*ANOVA was used to compare across all groups (asterisks indicate statistical significance across groups). Welch's t-test was also used to compare between groups. The following labelling convention was used to indicate statistical significance as determined by p-values.:*

** = $p < 0.05$*

*** = $p < 0.01$*

**** = $p < 0.001$*

***** = $p < 0.0001$*

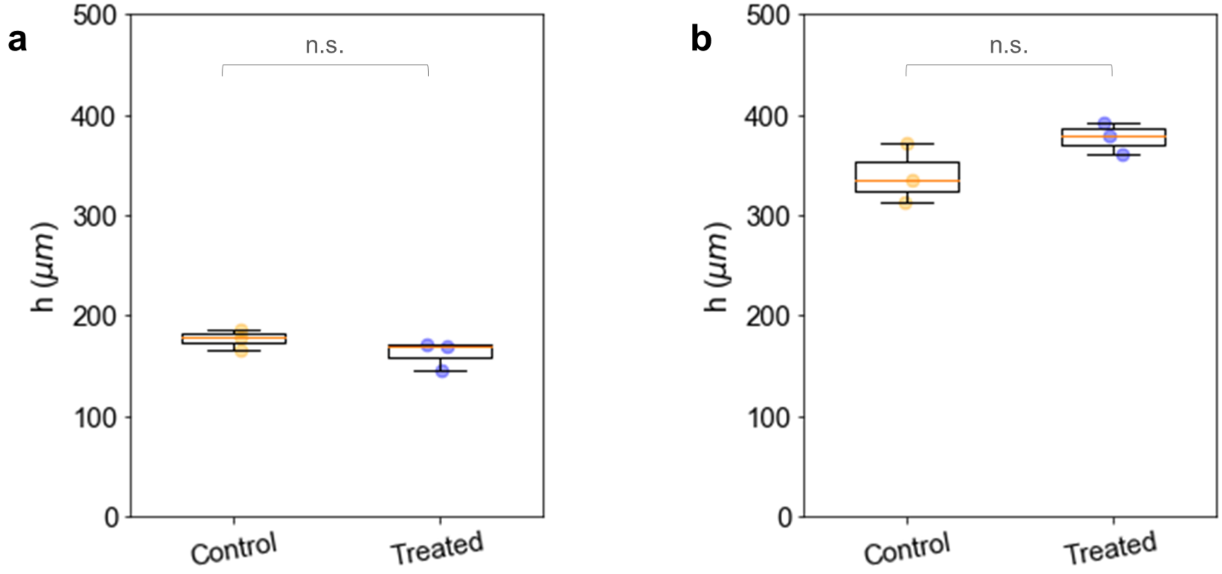


**Figure S7: (a)** Acridine orange incubation (at standard concentrations used for RBC staining as per the protocol outlined in the Methods, in the standard medium and levitation time) does not have a significant effect on levitation height of RBCs (uninfected). Control, with PBS (n = 3), and acridine orange-treated (n = 3) samples were compared. No significant difference was found between the groups, compared using Welch's t-test (p = 0.249).

**(b)** Acridine orange incubation (at standard concentrations used for RBC staining as per the protocol outlined in the Methods, in the standard medium and levitation time) does not have a significant effect on levitation height of WBCs (nucleated). Control, with PBS (n = 3), and acridine orange-treated (n = 3) samples were compared. No significant difference was found between the groups, compared using Welch's t-test (p = 0.144).

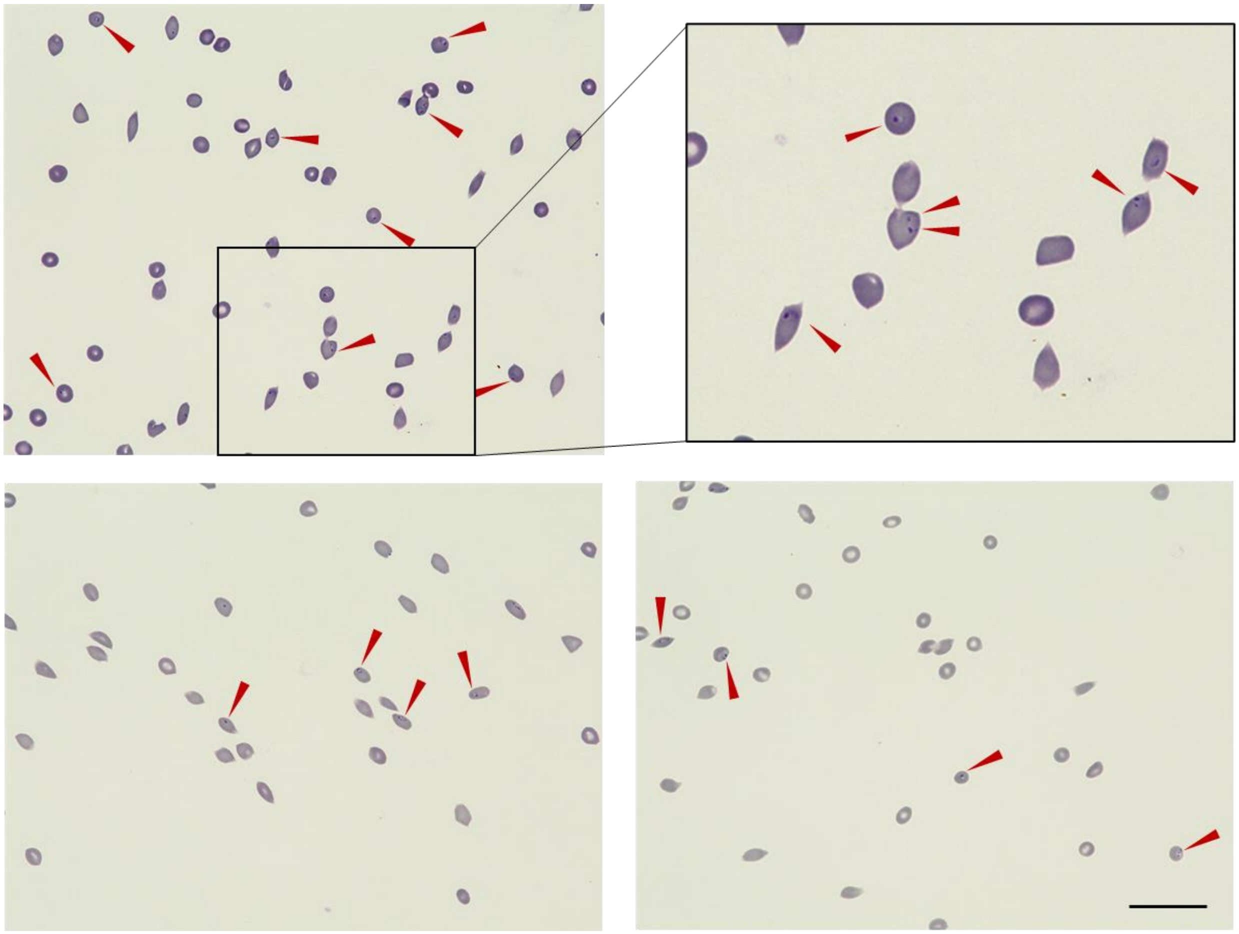

**Figure S8.** Three example images of Field-stained smears of ring-stage synchronized 3D7 cultures, corresponding to samples that were used in levitation experiments, and one inset focusing on a small selection of cells. A high-parasitemia sample (>10%) was selected in order to show multiple examples of stained ring-stage infected erythrocytes. In some cases, erythrocytes are infected with multiple ring-stage parasites. Some examples of the stained ring structures have been marked with red arrows for clarity. Scale bar = 50 µm.

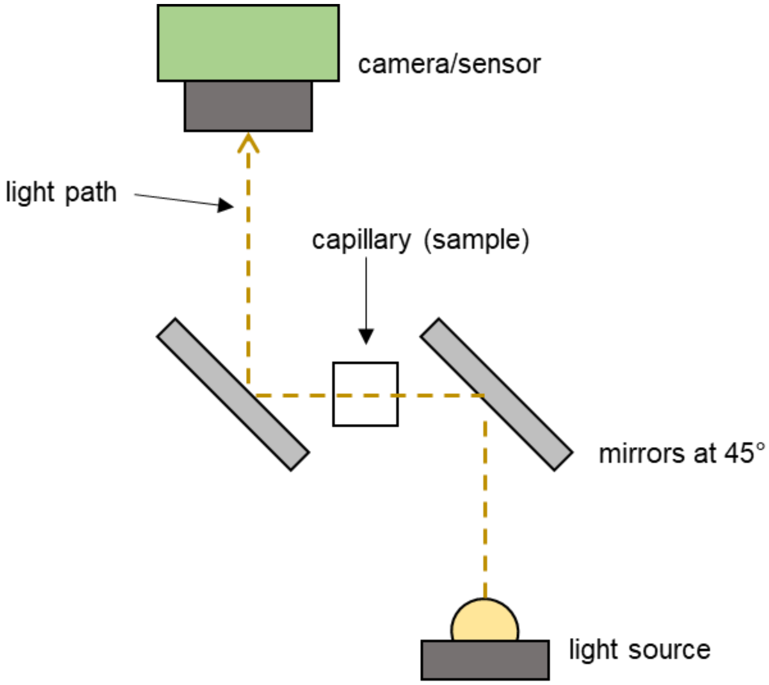


**Figure S9:** Use of mirrors to angle light for a standard vertical light path microscopy setup.

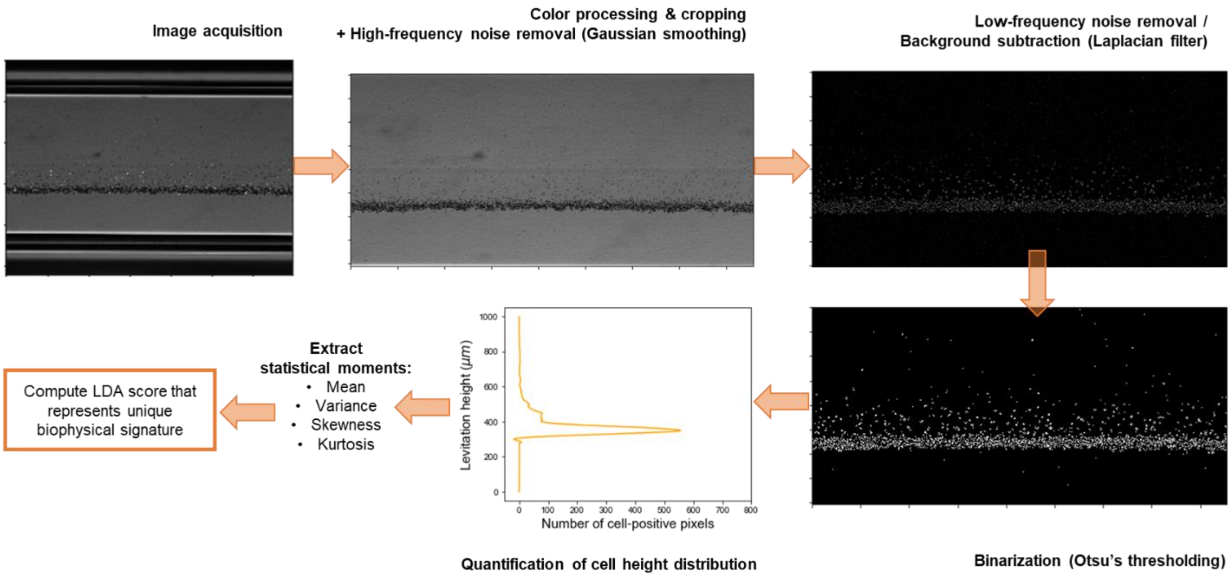


**Figure S10.** Workflow of image analysis of levitation images in custom OpenCV-based algorithm in python.

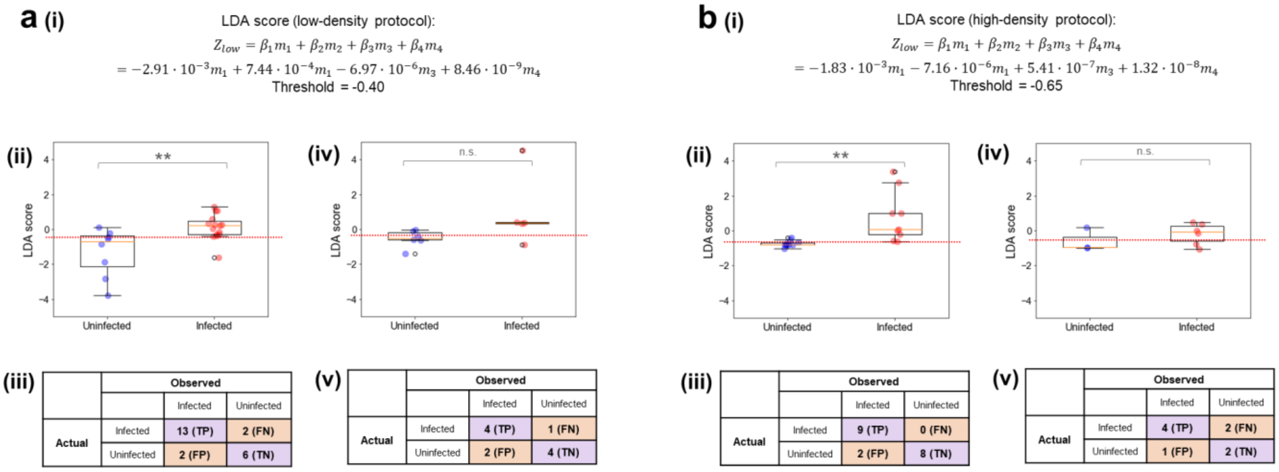


**Figure S11.** Performance of LDA score when applied to the dataset after splitting into training and test groups. These data are the same four statistical metrics quantified in Figure 4, but the LDA scoring was applied differently.

(**a**) Low-density protocol: the dataset (uninfected and infected samples) were randomly split into a training group (70%) and a test group (30%). Linear Discriminant Analysis (LDA) scoring was performed on the statistical metrics (mean, variance, skewness, and kurtosis) of the training group, to classify them into healthy or infected groups. **(i)** The resulting formula to calculate the LDA score as a recombination of the four metrics. **(ii)** Box and whisker plots show distribution of LDA scores for each sample tested. The scores for the two groups were statistically significant according to Welch's t-test, with a p-value of $3.6 \cdot 10^{-3}$. **(iii)** A confusion matrix classifying each sample by its score, by applying a threshold of optimal separation (-0.40). **(iv)** The same scoring formula applied to the test group (which was not seen by the algorithm during training). Box and whisker plots show the resulting distribution of LDA scores for each sample tested. The scores for the two groups were not statistically significant according to Welch's t-test, with a p-value of 0.12. **(iii)** A confusion matrix classifying each sample by its score, by applying the same threshold of optimal separation for the training group (-0.40).

(**b**) High-density protocol: the dataset (uninfected and infected samples) were randomly split into a training group (70%) and a test group (30%). Linear Discriminant Analysis (LDA) scoring was performed on the statistical metrics (mean, variance, skewness, and kurtosis) of the training group, to classify them into healthy or infected groups. **(i)** The resulting formula to calculate the LDA score as a recombination of the four metrics. **(ii)** Box and whisker plots show distribution of LDA scores for each sample tested. The scores for the two groups were statistically significant according to Welch's t-test, with a p-value of $4.8 \cdot 10^{-3}$. **(iii)** A confusion matrix classifying each sample by its score, by applying a threshold of optimal separation (-0.65). **(iv)** The same scoring formula applied to the test group (which was not seen by the algorithm during training). Box and whisker plots show the resulting distribution of LDA scores for each sample tested. The scores for the two groups were not statistically significant according to Welch's t-test, with a p-value of 0.41. **(iii)** A confusion matrix classifying each sample by its score, by applying the same threshold of optimal separation for the training group (-0.65).

Notes: TP = true positive, FP = false positive, FN = false negative, TN = true negative values.

Bar charts show means of the calculated statistical parameters for the sampled healthy and infected groups, with error bars indicating the standard error of the mean.

**Supplementary Table 1: List of abbreviations and symbols used**

| | |
|---|---|
| RBC | Red blood cell (erythrocyte) |
| Hb | Hemoglobin |
| *P. falciparum* | *Plasmodium falciparum* |
| $\chi$ | *'chi'*, magnetic susceptibility |
| $\rho$ | *'rho'*, density |
| *h* | Height (vertical axis, to represent levitation height) |
| $NaNO_2$ | Sodium nitrite |
| PBS | Phosphate buffered solution |
| SLO | Streptolysin O |
| LDA | linear discriminant analysis |
| ROC | Receiver operating characteristic |
| *d* | Height of channel (in meters) |
| *g* | Acceleration due to gravity, 9.8 $m \cdot s^{-2}$ |
| *p* | Permeability of free space, 0.01257 $m \cdot g \cdot s^{-2} \cdot A^{-2}$ |
| *B* | Surface magnetic field strength, in mT, or $g \cdot s^{-2} \cdot A^{-1}$ |
| PMMA | Poly(methyl methacrylate) |
| ANOVA | Analysis of variance |
| uRBC | Uninfected RBC |
| iRBC | Infected RBC |
| ring-iRBC | ring-stage synchronized infected RBC |
| troph-iRBC | trophozoite-stage (i.e. more mature) infected RBC |
| AUC | Area under the curve |
| FPR | False positive rate |
| TPR | True positive rate |
| TP | True positive |
| FP | False positive |
| FN | False negative |
| TN | True negative |
| FDA | Food and Drug Administration |
| PVP | Polyvinylpyrrolidone |
| Hct | Hematocrit |
| BSA | Bovine serum albumin |
| RGB | Red-green-blue |
| TIF | Tag Image File (format) |
| Hz | Hemozoin |
| QPS | Quantitative Phase Spectroscopy |
| DHA | Dihydroartemisinin |
| WBC | White blood cells (leukocytes) |
