# Supplementary material for: Multiparametric biophysical profiling of red blood cells in malaria infection
Source: Commun Biol. 2021 Jun 8;4:697. doi: 10.1038/s42003-021-02181-3 (PMC8187722; doi:10.1038/s42003-021-02181-3)
Supplement: Supplementary file 3 — Description of Supplementary Files [file 42003_2021_2181_MOESM3_ESM.pdf]

Description of Additional Supplementary Files

File name: Supplementary Data 1

Description: The underlying numeric source data for all graphs is tabulated, organized by each figure in which the data appears, in an attached Excel spreadsheet.
