# Supplementary material for: Multiparametric biophysical profiling of red blood cells in malaria infection
Source: Commun Biol. 2021 Jun 8;4:697. doi: 10.1038/s42003-021-02181-3 (PMC8187722; doi:10.1038/s42003-021-02181-3)
Supplement: Supplementary file 5 — Reporting Summary [file 42003_2021_2181_MOESM5_ESM.pdf]

nature research

Corresponding author(s): Utkan Demirci

Last updated by author(s): Apr 18, 2021

Reporting Summary

Nature Research wishes to improve the reproducibility of the work that we publish. This form provides structure for consistency and transparency in reporting. For further information on Nature Research policies, see our Editorial Policies and the Editorial Policy Checklist.

Statistics

For all statistical analyses, confirm that the following items are present in the figure legend, table legend, main text, or Methods section.

n/a	Confirmed	
☐	☒	The exact sample size (*n*) for each experimental group/condition, given as a discrete number and unit of measurement
☐	☒	A statement on whether measurements were taken from distinct samples or whether the same sample was measured repeatedly
☐	☒	The statistical test(s) used AND whether they are one- or two-sided<br>*Only common tests should be described solely by name; describe more complex techniques in the Methods section.*
☒	☐	A description of all covariates tested
☐	☒	A description of any assumptions or corrections, such as tests of normality and adjustment for multiple comparisons
☐	☒	A full description of the statistical parameters including central tendency (e.g. means) or other basic estimates (e.g. regression coefficient) AND variation (e.g. standard deviation) or associated estimates of uncertainty (e.g. confidence intervals)
☐	☒	For null hypothesis testing, the test statistic (e.g. *F*, *t*, *r*) with confidence intervals, effect sizes, degrees of freedom and *P* value noted<br>*Give P values as exact values whenever suitable.*
☒	☐	For Bayesian analysis, information on the choice of priors and Markov chain Monte Carlo settings
☒	☐	For hierarchical and complex designs, identification of the appropriate level for tests and full reporting of outcomes
☒	☐	Estimates of effect sizes (e.g. Cohen's *d*, Pearson's *r*), indicating how they were calculated

Our web collection on statistics for biologists contains articles on many of the points above.

Software and code

Policy information about availability of computer code

Data collection	Zeiss ZEN, Blue edition, software was used to collect microscope image data.
Data analysis	All image analysis was carried out in (open source) Python 2.7 and the modules and algorithms used are described in detail in the Methods and the Supplement.

For manuscripts utilizing custom algorithms or software that are central to the research but not yet described in published literature, software must be made available to editors and reviewers. We strongly encourage code deposition in a community repository (e.g. GitHub). See the Nature Research guidelines for submitting code & software for further information.

Data

Policy information about availability of data

All manuscripts must include a data availability statement. This statement should provide the following information, where applicable:

- Accession codes, unique identifiers, or web links for publicly available datasets
- A list of figures that have associated raw data
- A description of any restrictions on data availability

In this paper, Figures 2, 3, and 4, and the supplementary information have associated raw data that are the raw images of levitation patterns from which cell heights have been extracted as per the described methods. The numerical features of the cell height distributions are included in the Supplementary data attachment, and original images are available upon request.

Field-specific reporting

Please select the one below that is the best fit for your research. If you are not sure, read the appropriate sections before making your selection.

- [x] Life sciences
- [ ] Behavioural & social sciences
- [ ] Ecological, evolutionary & environmental sciences

For a reference copy of the document with all sections, see nature.com/documents/nr-reporting-summary-flat.pdf

Life sciences study design

All studies must disclose on these points even when the disclosure is negative.

Sample size	Power calculations were made based on preliminary data to determine minimum sample size, taking into account sample availability.
Data exclusions	No data were excluded.
Replication	Experiments were replicated and were generally found to be reproducible, with a minority of exceptions that have been noted in the paper and hypothesized to be due to naturally occurring biological variation as described there in further detail.
Randomization	Samples were allocated as uninfected or infected (by culture), and infection stages were separated by sorbitol synchronization (to select for rings) or by timepoints (confirmed by morphological characteristics in microscopy).
Blinding	Blinding was not relevant to this study because it was an exploratory study and because the same analysis methods were applied to images from different categories.

Reporting for specific materials, systems and methods

We require information from authors about some types of materials, experimental systems and methods used in many studies. Here, indicate whether each material, system or method listed is relevant to your study. If you are not sure if a list item applies to your research, read the appropriate section before selecting a response.

Materials & experimental systems

n/a	Involved in the study	
x		Antibodies
	x	Eukaryotic cell lines
x		Palaeontology and archaeology
x		Animals and other organisms
x		Human research participants
x		Clinical data
x		Dual use research of concern

Methods

n/a	Involved in the study	
x		ChIP-seq
x		Flow cytometry
x		MRI-based neuroimaging

Eukaryotic cell lines

Policy information about cell lines

Cell line source(s)	3D7 and W2 strains of Plasmodium falciparum were cultured in human erythrocytes.
Authentication	N/A
Mycoplasma contamination	Cell lines were not tested for mycoplasma contamination.
Commonly misidentified lines (See ICLAC register)	None were used.
