# Supplementary material for: Multiparametric biophysical profiling of red blood cells in malaria infection
Source: Commun Biol. 2021 Jun 8;4:697. doi: 10.1038/s42003-021-02181-3 (PMC8187722; doi:10.1038/s42003-021-02181-3)
Supplement: Supplementary file 6 — Software Policy Checklist [file 42003_2021_2181_MOESM6_ESM.pdf]

nature research

Corresponding author(s): Dr. Utkan Demirci

Code and Software Submission Checklist

Prior to submitting your work to Nature Research, we strongly recommend that you ask at least one colleague who is unfamiliar with your software to install the tool(s), follow the instructions, and provide feedback. This process will help ensure that reviewers will also be able to run your software.

You must submit all required content as a single zip file prior to peer review or provide a link where editors and reviewers can access all required content.

Required content

- [x] Compiled standalone software and/or source code
- [x] A small (simulated or real) dataset to demo the software/code

A README file that includes:

1. System requirements
   - [x] All software dependencies and operating systems (including version numbers)
   - [x] Versions the software has been tested on
   - [x] Any required non-standard hardware
2. Installation guide
   - [x] Instructions
   - [x] Typical install time on a "normal" desktop computer
3. Demo
   - [x] Instructions to run on data
   - [x] Expected output
   - [x] Expected run time for demo on a "normal" desktop computer
4. Instructions for use
   - [x] How to run the software on your data
   - [ ] (OPTIONAL) Reproduction instructions

   We encourage you to include instructions for reproducing all the quantitative results in the manuscript.

Additional information

Describe your software's license for use. We strongly recommend using a license approved by the Open Source Initiative.

A GNU General Public License Version 3.0, which is approved by the Open Source Initiative, permits broad use on the condition that any modifications are also made available open source.

Provide a link to the code in an open source repository (when available).

https://doi.org/10.6084/m9.figshare.14445223.v1

Your manuscript should include a complete, detailed description of the code's functionality (i.e. pseudocode).
Please indicate where this is found:

- [ ] Main text
- [x] Methods section
- [ ] Elsewhere (specify):

Examples of well-structured software packages

1. https://github.com/neurodata-papers/MGC
2. https://github.com/neurodata-papers/LOL
3. https://www.nature.com/nbt/journal/v34/n6/abs/nbt.3569.html#supplementary-information
4. https://www.nature.com/nature/journal/v548/n7669/full/nature23463.html#extended-data
https://github.com/yasharhezaveh/Ensai
5. https://www.nature.com/nbt/journal/v34/n11/full/nbt.3685.html#supplementary-information
https://github.com/IFIproteomics/LFQbench
